# Dysbiosis in the Pathogenesis of Atopic Dermatitis

**DOI:** 10.1111/1346-8138.70191

**Published:** 2026-02-20

**Authors:** Hiroki Okamoto, Yuumi Nakamura

**Affiliations:** ^1^ Department of Dermatology The University of Osaka, Graduate School of Medicine Osaka Japan; ^2^ Cutaneous Allergy and Host Defense, Immunology Frontier Research Center The University of Osaka Osaka Japan

**Keywords:** atopic dermatitis, dysbiosis, microbiome, quorum‐sensing, *Staphylococcus aureus*

## Abstract

Atopic dermatitis (AD) is a chronic inflammatory skin disease characterized by epidermal barrier dysfunction and immune dysregulation. Recent research highlights cutaneous dysbiosis as a critical factor in its pathogenesis. In this review, we summarize the interplay between the skin microbiota and host immunity, contrasting the homeostatic state with the dysbiosis in AD. In healthy skin, resident microbial communities, including coagulase‐negative staphylococci and *Cutibacterium acnes*, contribute to immune education and pathogen defense. In AD, this equilibrium is disrupted, leading to a state of functional dysbiosis characterized not only by reduced microbial diversity and the predominance of *Staphylococcus aureus* but also by the loss of protective commensal functions. The virulence of *S. aureus* is pivotal, with its accessory gene regulator (Agr) quorum‐sensing system driving the expression of toxins like δ‐toxin, which exacerbates type 2 inflammation and barrier defects. Crucially, colonization in early life with *S. aureus* strains possessing a functional Agr system is strongly associated with an increased risk of subsequent AD development. This understanding has prompted a paradigm shift in therapeutic strategies. Recognizing the limitations of traditional broad‐spectrum antimicrobials, which can worsen dysbiosis, novel approaches now focus on restoring microbial balance. These include bacteriotherapy using beneficial commensal strains to competitively inhibit *S. aureus* , quorum‐quenching agents, and preventive skincare interventions initiated in infancy to foster a healthy microbiome. A deeper comprehension of these host‐microbe and microbe‐microbe interactions is essential for optimizing these promising microbiome‐targeted therapies for AD.

## 1 Introduction

The skin is the largest organ of the human body and plays a crucial role in maintaining overall health. It serves as a physical barrier that protects the body from harmful microorganisms and substances such as bacteria, viruses, and other pathogens. At the same time, the skin surface harbors a diverse community of microorganisms, including bacteria, fungi, and viruses, that coexist and interact to form the cutaneous microbiota [1].

Since the mid‐2000s, advances in next‐generation sequencing technologies, particularly 16S rRNA‐based microbiome analysis, have accelerated research on microbial communities across various body sites, including the gut, skin, and oral cavity. These studies have revealed that resident microbial communities are not merely coexisting entities but indispensable components for immune system development and tissue homeostasis. In contrast, dysbiosis, defined as microbial imbalance, has been implicated in the onset and progression of various pathological conditions, including inflammatory and allergic diseases [2, 3]. Atopic dermatitis (AD) has long been recognized as a prototypical chronic inflammatory skin disease; however, it is now increasingly recognized that epidermal barrier dysfunction and microbial dysbiosis are critical components involved in its pathogenesis [4, 5, 6, 7]. Epidemiologically, AD affects approximately 15%–20% of children and 2%–10% of adults worldwide, substantially impairing quality of life. Its prevalence continues to rise globally [1, 8]. The pathogenesis of AD is multifactorial, involving filaggrin gene mutations that compromise barrier integrity, environmental exposures that promote allergen sensitization, skewed immune responses dominated by Th2 type pathways, and alterations in the cutaneous microbiota [9, 10, 11]. This complex interplay among these elements underlies the chronic and relapsing inflammatory nature of the disease. In this review, we focus on the skin microbiota and provide an overview of how changes in its composition and dynamics contribute to the pathogenesis of AD. Particular attention is given to the roles of *Staphylococcus aureus* predominance and reduced microbial diversity in driving the onset and exacerbation of AD, as well as to therapeutic strategies that target these microbial changes.

## 2 Skin Microbiota and Immune Homeostasis in Health

The skin serves as a physical barrier against the external environment, preventing the penetration of harmful substances and blocking the loss of water and essential molecules, thereby playing a crucial role in systemic homeostasis. However, the skin is not merely a static protective surface; recent studies have revealed that it represents a dynamic immunological environment in which immune responses are continuously regulated through interactions with resident microbial communities. Adult human skin, including appendages such as hair follicles, eccrine ducts and sebaceous glands, covers a surface area of more than 30 square meters and harbors approximately 10^6^ bacteria per square centimeter, comprising at least 40 major bacterial taxa [12, 13, 14, 15]. Compared with the gut microbiome, the skin microbiome exhibits significantly lower diversity [16].

Unlike the intestinal mucosa, which provides a humid and nutrient‐rich environment that favors bacterial colonization, mammalian skin constitutes a relatively dry and nutrient‐poor habitat. As a result, its colonization is dominated by bacterial groups that have evolved to thrive in this niche, particularly members of the phyla Firmicutes and Actinobacteria. Many of these taxa harbor lipid‐modifying enzymes such as triacylglycerol hydrolases, cholesterol‐modifying enzymes, and ceramidases, which enable them to exploit skin lipids as a key nutrient source [17]. The predominant genera colonizing the skin include *Staphylococcus, Cutibacterium, Corynebacterium, Micrococcus*, and *Acinetobacter* [16]. Their relative abundance varies according to individual host characteristics such as age and sex, as well as by anatomical site, with sebaceous, moist, and dry regions each harboring distinct microbial communities. Sites with similar physiological characteristics tend to support similar microbial assemblages, reflecting adaptation to local factors including temperature, moisture, pH, and lipid content [14, 18]. For example, sebaceous regions, such as the glabella, external auditory canal, chest, and back, are predominantly colonized by members of the genera *Cutibacterium* and *Staphylococcus*. In contrast, intertriginous regions that are prone to moisture accumulation, including the inguinal folds, antecubital fossae, and popliteal fossae, are dominated by *Corynebacterium* species. Meanwhile, dry regions such as the volar forearm, hypothenar palm, and buttocks show increased representation of specific lineages within the order Flavobacteriales and β‐proteobacterial taxa, resulting in greater microbial diversity [14].

## 3 Developmental Dynamics of the Skin Microbiota and Host Immunity

The skin microbiota is established early in life, and its initial composition is strongly influenced by delivery mode. Vaginally delivered infants acquire microbial communities resembling the maternal vaginal microbiota, whereas those born by cesarean section harbor microbiota more similar to maternal skin [19]. With aging, the composition undergoes substantial shifts. During puberty, for instance, sebaceous gland development drives significant remodeling of microbial communities in sebaceous regions. At Tanner stages I‐III, diverse taxa such as *Streptococcus* and Gram‐negative genera including *Moraxella, Haemophilus*, *and Neisseria* are detectable. By Tanner stages IV–V, however, these taxa are largely absent and lipidophilic genera such as *Cutibacterium, Corynebacterium*, and *Turicella* predominate [20]. Longitudinal studies in healthy adults over 1 year have shown that during adulthood, the skin microbiota stabilizes at the species level, maintaining a relatively consistent composition across seasons [21, 22].

Importantly, the skin microbiota comprises both resident microorganisms that form the stable core microbiome and transient microorganisms acquired from the environment that persist only for hours to days [23]. The core microbiome coexists symbiotically with the host and plays a central role in maintaining skin homeostasis [24, 25]. Commensal microbes acquired during these developmental processes actively contribute to host immune education. *Staphylococcus epidermidis*, for example, recruits CD4^+^ regulatory T cells to the skin during the neonatal period, thereby establishing tolerance to resident commensals [24, 26]. In contrast, IL‐17A^+^ CD8^+^ T cells reinforce protective immunity against pathogens such as *Candida albicans* and *Leishmania major* [24, 27]. In addition, coagulase‐negative staphylococci (CoNS), including *S. epidermidis* and *Staphylococcus hominis*, produce antimicrobial peptides (AMPs) that inhibit colonization by pathogenic *S. aureus* and stimulate keratinocytes to produce β‐defensins and cathelicidins, thereby enhancing innate immunity [28, 29, 30, 31, 32]. Although keratinocytes intrinsically produce AMPs, their basal expression remains relatively low under steady‐state conditions. Despite this, healthy skin remains largely infection‐free, a phenomenon attributed to resident microbes producing antimicrobial substances that establish a chemical barrier on the skin surface [30]. Furthermore, certain commensal bacteria enhance AMP expression and promote neutrophil recruitment, reinforcing host defense against pathogens, such as *S. aureus* [33, 34]. Beyond these effects, *S. epidermidis* has been shown to modulate cytokine networks by inducing IL‐1α expression while suppressing IL‐1 receptor antagonist expression in the epidermis [35]. This modulation promotes IL‐17A and IFNγ production by resident CD8^+^ T cells, strengthening innate immune defense against pathogens [24]. Such host–microbe interactions are critical for barrier maturation and wound healing [36, 37] and collectively sustain skin health and immune balance [35].

Overall, skin commensals exert dual roles: they foster immune tolerance toward resident microorganisms while simultaneously activating protective immunity against invading pathogens. The neonatal period represents a critical window for immune education through early microbial encounters, whereas in adulthood, stable crosstalk between the host and resident microbes supports barrier integrity and infection control. Disruption of this equilibrium, namely skin microbiota dysbiosis, may therefore act not only as a downstream consequence of disease but also as a contributing factor in the pathogenesis of skin disorders, including AD.

## 4 Microbial Shifts and Dysbiosis in Atopic Dermatitis

Dysbiosis, defined as an imbalance of the microbial community, is widely recognized in inflammatory diseases, including those of the skin [38]. It is typically characterized by reduced microbial diversity, often accompanied by the dominance of particular species and shifts in overall microbial composition. Recent evidence indicates that the skin microbiome of patients with AD exhibits marked compositional and functional alterations compared with that of healthy individuals, with particular emphasis on the association between cutaneous dysbiosis and *S. aureus* colonization [3]. The presence of *S. aureus* on AD lesions was first reported in the 1960s [39], and this link was notably strengthened in 1974 when large numbers of *S. aureus* were reported on lesional skin even in patients without clinical signs of infection [40]. These early, culture‐based findings were later substantiated by modern sequencing studies, which confirmed both reduced microbial diversity and elevated relative abundance of *Staphylococcus*, particularly *S. aureus* [5, 7]. Meta‐analyses have shown that approximately 70% of AD patients harbor *S. aureus* on lesional skin, compared with 39% on nonlesional or healthy skin, and its abundance correlates positively with disease severity [41, 42]. Longitudinal pediatric studies further demonstrate that *S. aureus* density increases during disease flares and decreases during remission, underscoring its dynamic role in AD exacerbation [43]. Dysbiosis involves not only the overgrowth of pathogens but also the loss or distortion of commensal‐derived protective functions. In healthy skin, *S. epidermidis* promotes immune tolerance and barrier defense, for instance by recruiting CD4^+^ regulatory T cells and boosting antimicrobial activity [24, 26, 33, 34, 35]. In AD, however, some *S. epidermidis* strains express the cysteine protease EcpA, which disrupts the barrier and promotes inflammation [44], illustrating a strain‐dependent functional “duality” and underscoring how commensals can be either protective or pathogenic depending on strain‐specific traits. Overall, dysbiosis in AD can be conceptualized as a multifaceted phenomenon encompassing (i) reduced microbial diversity, (ii) overrepresentation of *S. aureus* , and (iii) loss of protective commensal functions. These alterations not only represent downstream consequences of skin inflammation but may also act as active drivers of AD pathogenesis, reinforcing a vicious cycle between microbial imbalance and immune dysregulation.

## 5 Immune Dysregulation Driven by Dysbiosis

The consequences of these microbial shifts extend far beyond a simple change in composition. Mounting evidence demonstrates that dysbiosis is not a passive consequence of inflammation but an active driver of the immune dysregulation and barrier deterioration that perpetuate the disease [4, 9]. At the core of this dysregulated environment lies the amplification of Th2 type immune responses. Keratinocyte‐derived cytokines such as thymic stromal lymphopoietin (TSLP) promote Th2 cell differentiation and Langerhans cell activation. Subsequently, Th2 cells secrete IL‐4, IL‐13, and IL‐31, which directly stimulate sensory neurons to induce pruritus [45] and can also compromise barrier integrity by modulating keratinocyte function, leading to reduced expression of key barrier proteins [9, 10]. In contrast, during the chronic phase, type 1 cytokines such as IFNγ are upregulated, and additional T cell subsets including Th22 and Th17 cells and their associated IL‐23 pathways become involved [4, 46, 47]. This transition underscores the evolving nature of AD immunopathology, in which acute Th2 responses evolve into a more complex immune dysregulation. Importantly, dysbiosis of the skin microbiota is now recognized as a critical environmental factor that exacerbates this immune imbalance. Loss of beneficial commensal functions weakens antimicrobial defense and immune regulation, while the predominance of *S. aureus* provides potent stimuli for Th2 amplification and barrier impairment [6, 48, 49]. This pathogenic role of *S. aureus* has been consistently validated and further elucidated through diverse in vivo studies. Indeed, studies in various AD mouse models further support this notion. For instance, filaggrin‐deficient flaky tail (*Flgft/ft*) mice, which exhibit impaired skin barrier function akin to human AD, show increased *S. aureus* abundance directly correlating with elevated levels of Th2‐associated cytokines such as IL‐4, IL‐13, and IL‐22 [50]. Similarly, ADAM17‐deficient mice, displaying eczematous dermatitis and skin barrier dysfunction, develop dysbiosis with increased *S. aureus* colonization, and antibiotic treatment significantly reduces both dysbiosis and skin inflammation [51]. These findings underscore the critical link between impaired barrier and susceptibility to *S. aureus* overgrowth. Furthermore, genetically engineered mouse models overexpressing IL‐4 in keratinocytes spontaneously develop *S. aureus* colonization on the skin [52], illustrating how a Th2 type skin inflammation can itself promote colonization. Taken together, these models support a bidirectional framework in which primary barrier or immune defects can promote dysbiosis, and the resulting microbial imbalance can further amplify inflammation and barrier deterioration. This microbial imbalance interacts with host immunity to sustain a vicious cycle of inflammation, pruritus, and barrier disruption. Therefore, microbial imbalance in AD should not be regarded merely as a consequence of skin inflammation but rather as a central factor that modulates immune responses, drives chronicity, and predisposes to disease exacerbation.

## 6 *Staphylococcus aureus* in AD: Virulence Mechanisms and Host Disruption

The prominent role of *S. aureus* in exacerbating AD is largely attributed to its array of diverse virulence factors that directly interact with host tissues and immune responses. These factors collectively contribute to epidermal barrier dysfunction, inflammation, and immune dysregulation, thereby perpetuating the chronic nature of AD. Among these, certain key components have been extensively studied for their individual and coordinated effects. Specifically, molecules of *S. aureus* such as α‐hemolysin (Hla), lipoteichoic acid (LTA), peptidoglycan (PGN), and staphylococcal protein A (SPA) are known to be involved in inflammatory reactions of the skin. For instance, Hla disrupts keratinocytes and compromises the epidermal barrier [53]. LTA and PGN function as Toll‐like receptor ligands that activate inflammatory signaling [54], as previously reported in various studies [54, 55]. Although SPA interferes with opsonization and enhances IL‐18 production, promoting immune evasion [56], topical application of SPA alone does not induce dermatitis in mice [57].

These findings indicate that while individual factors can injure tissue and perturb immunity, their pathogenic effect in AD is context dependent and often insufficient in isolation. Instead, it is more plausible that persistent colonization and chronic inflammation are maintained by the coordinated, population‐density‐dependent control of toxins and proteases acting in concert. Accordingly, the accessory gene regulator (Agr) quorum‐sensing (QS) system is now regarded as an important factor of *S. aureus* pathogenicity in AD. Accumulation of autoinducing peptides (AIPs) activates the AgrC/AgrA two‐component system, which in turn drives transcription from the P2 and P3 promoters. P2 activation amplifies AIP production via upregulation of the *agr* operon, whereas P3 induces RNAIII, a regulatory RNA that controls the expression of multiple toxins and enzymes [58]. Among these Agr‐regulated products, δ‐toxin has emerged as a key factor in AD. It promotes mast cell degranulation and enhances IgE and IL‐4 responses, linking *S. aureus* colonization with type 2 inflammation [6]. Elevated δ‐toxin expression has been documented in isolates from AD lesions, and wild‐type strains producing δ‐toxin exacerbate dermatitis in murine models compared with toxin‐deficient mutants [6]. Furthermore, δ‐toxin promotes IL‐1α‐dependent epicutaneous sensitization, providing a mechanistic basis for the clinical association between AD and food allergy [59]. Phenol‐soluble modulins (PSMs), particularly PSMα3, also contribute to AD pathogenesis. PSMα3 induces keratinocyte cytotoxicity and triggers the release of alarmins such as IL‐1α and IL‐36α, thereby amplifying both Th2‐ and IL‐17‐mediated pathways [48]. These effects intensify inflammation, compromise barrier integrity, and facilitate further *S. aureus* colonization.

In addition to these toxins, *S. aureus* secretes several proteases that contribute to epidermal damage and symptom exacerbation. Among them, the V8 serine protease (SspA), whose production is also regulated by Agr‐QS, has recently gained attention for its role in pruritus. The V8 protease cleaves proteinase‐activated receptor 1 (PAR1) expressed on sensory neurons and keratinocytes, directly activating pruriceptive signaling pathways [60]. This proteolytic activation induces the release of neuropeptides and inflammatory mediators, thereby linking *S. aureus* colonization with itch and neuroimmune crosstalk in AD. These findings suggest that QS‐regulated proteolytic enzymes can also serve as key effectors amplifying inflammation and sensory dysfunction in AD.

**FIGURE 1:**
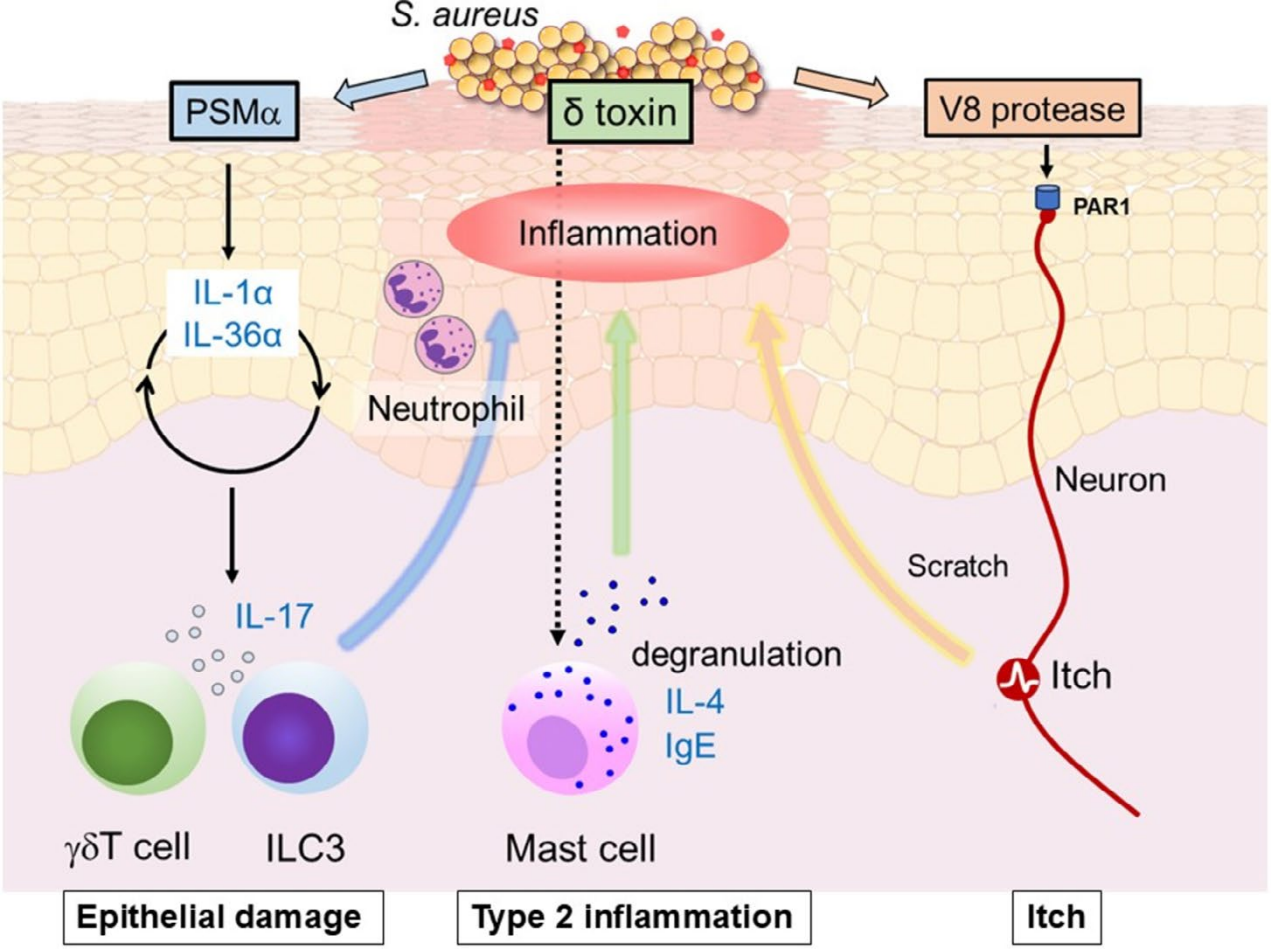
Agr‐regulated toxins and protease in AD. When *S. aureus* colonizes the skin surface, it activates the quorum‐sensing system. Upon detecting PSMα, keratinocytes release IL‐1α and IL‐36α as a result of epithelial damage, which in turn promote the induction of γδ T cells and ILC3s that produce IL‐17. IL‐17 recruits neutrophils, thereby eliciting a protective immune response against bacteria. δ‐toxin triggers mast‐cell degranulation, inducing IL‐4 and IgE, which contribute to the development of type 2 inflammation. The V8 protease from *S. aureus* directly activates pruriceptive sensory neurons by cleaving proteinase‐activated receptor‐1 (PAR1), leading to itch.

In summary, both QS‐dependent and QS‐independent virulence mechanisms act in concert to amplify Th2 inflammation, trigger alarmin release, impair host defense, and directly influence neuronal pathways. Thus, microbial imbalance in AD should be regarded not only as a secondary effect but also as an active driver of immune dysregulation and barrier disruption (Figure 1).

## 7 Early‐Life Microbial Exposure and Risk of AD

**FIGURE 2:**
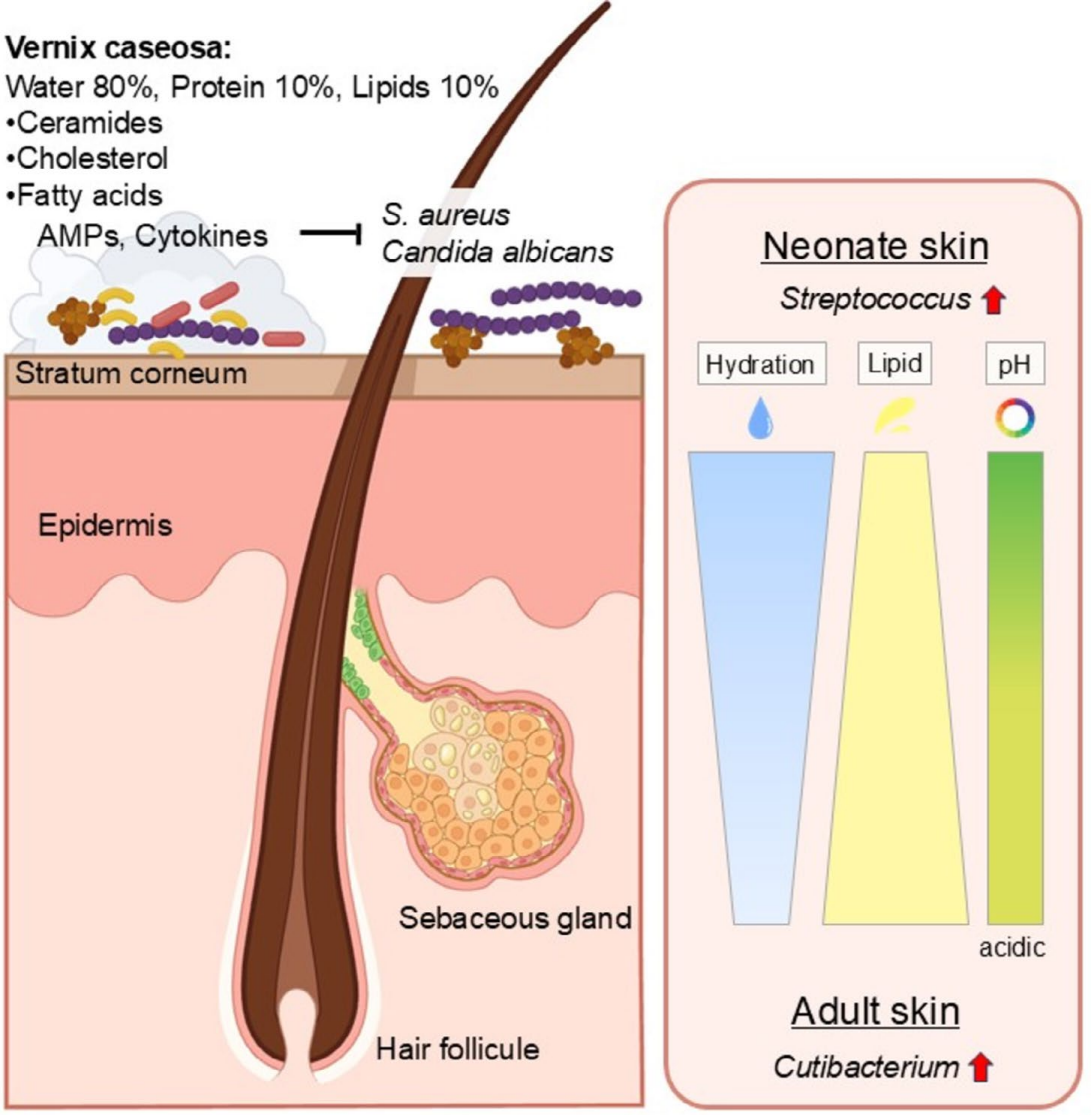
Vernix caseosa and neonatal skin microbiome. Vernix caseosa consists of 80% water, 10% protein, and 10% lipids, which include ceramides, cholesterol, and fatty acids. It also contains cytokines and antimicrobial peptides (AMPs). These components inhibit pathogens like *S. aureus* and *C. albicans* . Furthermore, the skin environment characterized by higher pH and lower lipid levels favors the genus *Streptococcus* colonization over lipophilic *Cutibacterium* species.

**FIGURE 3:**
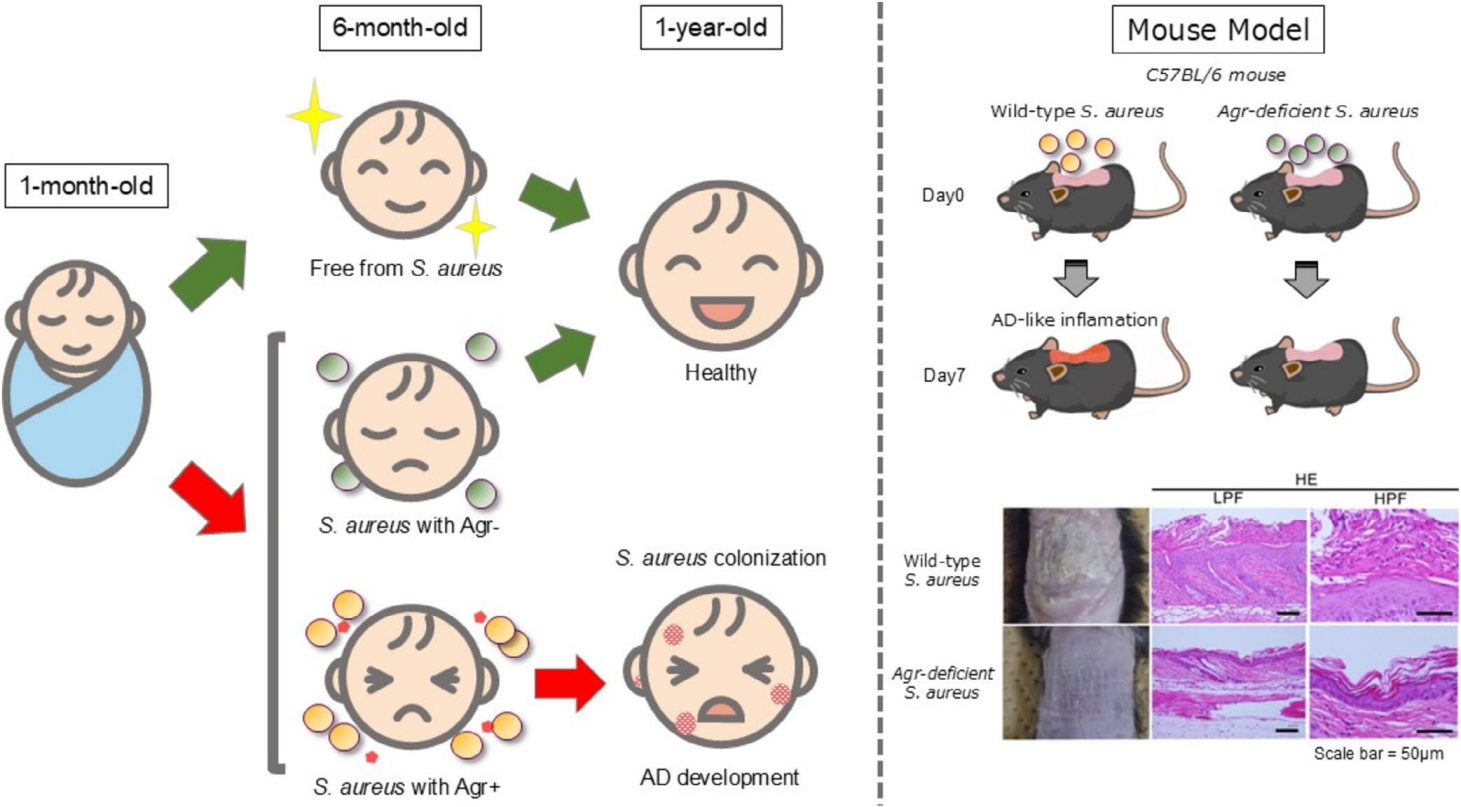
The role of the Agr‐QS system in *S. aureus* colonization in infants with AD. Infants colonized with *S. aureus* at 6 months of age exhibited a significantly higher risk of developing AD. Loss‐of‐function mutations in the *agr* locus were identified in *S. aureus* strains isolated from healthy infants who did not develop AD, but not in strains from infants who developed AD. Findings from murine models further demonstrated that the Agr‐QS system plays a crucial role in driving AD‐like inflammation. Photographs and histological images of mice are adapted from reference [49]. HE, Hematoxylin–Eosin Stain; HPF, high power field; LPF, low power field.

At birth, the skin represents the first organ exposed to the external environment, yet neonates are covered in the vernix caseosa. Vernix consists of approximately 80% water, 10% protein and 10% lipids, including ceramides, cholesterol and free fatty acids, and contains cytokines (IL‐1, IL‐1β, IL‐6, IL‐8, TNF‐α) as well as AMPs such as LL‐37, lysozyme, psoriasin, lactoferrin and α‐defensins, which are thought to inhibit colonization by pathogens including *S. aureus* and *Candida albicans* [61]. Compared with adult skin, infant skin is more hydrated, has a higher pH, and contains lower levels of lipids and sebum [62]. Microbiome analyses indicate that members of the genus *Streptococcus* thrive under conditions with higher pH and lower lipid levels, whereas lipophilic *Cutibacterium* species preferentially colonize acidic, lipid‐rich environments. By 1 year of age, correlations have been observed between the relative abundance of specific taxa and local skin pH or moisture, consistent with the ecological preferences of these organisms [63] (Figure 2). Building on this understanding of early skin microbiota, an important question is whether early‐life skin dysbiosis may serve as a biomarker or target for interventions to prevent the “allergic march,” in which allergic diseases accumulate sequentially. Early microbial exposures are crucial for shaping immune development and determining susceptibility to allergic disorders. AD commonly manifests in infancy and longitudinal birth cohort studies have provided key insights into the association between *S. aureus* colonization and AD onset. In a Japanese birth cohort of 268 infants, approximately 45% were colonized with *S. aureus* at 1 month of age, but this was not associated with AD incidence at 1 year. By contrast, colonization at 6 months was significantly associated with increased AD risk [49]. Whole‐genome sequencing of *S. aureus* strains revealed that isolates from infants who remained free of AD often carried loss‐of‐function mutations in the Agr‐QS locus, resulting in impaired QS signaling and poor persistence on the skin. In contrast, strains from infants who developed AD carried intact Agr systems, suggesting that a functional Agr‐QS circuit promotes stable colonization and drives AD pathogenesis [49]. Murine models of epicutaneous colonization further confirmed that Agr‐dependent *S. aureus* induces AD‐like inflammation and barrier disruption [49]. While most data support a pathogenic role for Agr‐QS, conflicting results have been reported. In a human skin xenograft model using SCID mice, Agr‐deficient USA300 MRSA strains achieved higher burdens than wild‐type strains [64]. This discrepancy may reflect differences in host immune competence. These findings suggest that a functional Agr‐QS system gives *S. aureus* an advantage in immunocompetent hosts, such as infants who are prone to AD. In contrast, under immunodeficient conditions or in other clinical contexts, Agr‐QS activity may not be as beneficial. In fact, reduced QS activity has been linked to stronger biofilm formation and greater antibiotic resistance, features that help *S. aureus* persist in hospital‐associated infections [65, 66]. In summary, early‐life microbial exposure represents a critical determinant of AD risk. Colonization with *S. aureus* strains possessing a functional Agr‐QS system constitutes a critical window for intervention, during which microbial cues may skew immune development toward disease. These insights highlight the potential for preventive strategies aimed at modulating early microbial colonization or targeting QS‐dependent virulence pathways (Figure 3).

## 8 Microbe–Microbe Interactions: Commensal Defense and Pathogen Competition

The cutaneous microbiota is shaped by continuous interactions among microbial species that determine colonization dynamics and ecological balance. Commensal bacteria not only compete with pathogens for space and nutrients but also secrete inhibitory molecules that restrict pathogenic expansion. In the context of AD, disruption of this equilibrium diminishes these protective mechanisms, allowing opportunistic species such as *S. aureus* and certain lineages of *S. epidermidis* to proliferate [5, 43]. Bacterial interactions can be either competitive or cooperative, and both are critical in influencing microbial community structure. The Agr‐QS system, extensively discussed in the previous section as a key determinant of *S. aureus* pathogenicity, also plays a crucial role in these interbacterial communications and competitive advantages. Although interspecies bacterial cell‐to‐cell communication in the human skin remains incompletely understood, *S. aureus* has been studied extensively as a model organism due to its Agr‐QS system and other virulence‐associated pathways. Importantly, commensal CoNS, such as *S. epidermidis* and *S. hominis* , produce AMPs that directly suppress *S. aureus* growth and colonization. Beyond AMP production, *S. epidermidis* secretes the serine protease Esp, which disrupts biofilm formation, a crucial mechanism for *S. aureus* persistence and epithelial colonization, thereby providing a dual defensive strategy [67]. Recent studies have identified additional commensal‐derived antimicrobial substances with potential therapeutic applications. For example, *Staphylococcus lugdunensis* produces the antimicrobial peptide lugdunin, which efficiently suppresses the growth of *S. aureus* and is notable for its low propensity to induce resistance, highlighting its promise against antibiotic‐resistant strains [31]. Similarly, fengycins, lipopeptides produced by Bacillus species, inhibit staphylococcal colonization and are under investigation as candidate probiotics [68]. The traditional understanding of certain bacterial species is also being revised. *Cutibacterium acnes* (formerly *Propionibacterium acnes* ), long regarded primarily as a pathogenic contributor to acne, has been reported to exert beneficial effects on skin homeostasis. Specifically, its metabolic activity contributes to lipid metabolism on the skin surface, generating metabolites such as short‐chain fatty acids and other lipid derivatives that may enhance antimicrobial activity, reduce transepidermal water loss (TEWL) and strengthen barrier function [69, 70].

Beyond bacteria, nonbacterial microorganisms also contribute to AD pathogenesis. Colonization by fungi of the genus *Malassezia*, the most common commensal fungi on human skin, has been shown to increase with AD severity. Protein antigens derived from *Malassezia* have been implicated in exacerbating AD lesions by stimulating keratinocytes and immune cells to produce pro‐inflammatory cytokines, thereby linking fungal overgrowth with disease severity [71, 72]. Conversely, other studies have suggested potentially protective roles of *Malassezia* species. While the overall abundance of the genus *Malassezia* does not uniformly decrease in AD, the relative abundance of *M. globosa* has been reported to be significantly reduced in AD skin compared with healthy controls [73]. Notably, *M. globosa* secretes aspartyl proteases such as MgSAP1, which inhibit *S. aureus* biofilm formation without affecting bacterial viability, suggesting a capacity to antagonize pathogenic colonization [74, 75]. Overall, these findings underscore that microbe–microbe interactions on the skin are essential for maintaining microbial balance and protecting against pathogenic overgrowth. When these commensal defense mechanisms are disrupted, as in AD, dysbiosis not only facilitates the proliferation of *S. aureus* but also contributes to disease exacerbation through the loss of protective functions provided by commensals.

## 9 Therapeutic Strategies Targeting Microbial Dysbiosis in AD

With growing evidence that the skin microbiota contributes to both the exacerbation and onset of AD, therapeutic and preventive approaches targeting microbial balance, such as topical probiotics, bacteriotherapy and microbiome‐derived peptides, have gained increasing attention. Traditional eradication strategies, including systemic antibiotics and sodium hypochlorite bleach baths, have generally proven ineffective and may worsen dysbiosis while promoting antimicrobial resistance [76, 77, 78, 79]. These limitations underscore the need for more selective strategies that suppress *S. aureus* while preserving beneficial commensals. Therefore, the Agr‐QS system of *S. aureus* has attracted considerable attention as a potential therapeutic target. Studies have shown that Agr‐QS inhibitors can suppress *S. aureus* virulence and attenuate skin inflammation in murine models of inflammatory skin disease [80, 81]. Despite these promising findings, many attempts to target AgrA, AgrC, and other QS components have yielded limited efficacy in vivo, and in some cases, quorum‐quenching approaches paradoxically enhanced biofilm formation, raising concerns about their clinical applicability [82].

An alternative and increasingly promising strategy involves exploiting the natural competition between *S. aureus* and commensal CoNS. Distinct AIPs produced by commensal CoNS, particularly *S. epidermidis* , can antagonize *S. aureus* Agr‐QS signaling and thereby reduce toxin expression [83, 84]. Building on this principle, bacteriotherapy approaches have been developed. One example is *Staphylococcus hominis* A9 (ShA9), a commensal strain that produces lantibiotics with broad‐spectrum anti‐ *S. aureus* activity while sparing beneficial microbes such as *S. epidermidis* . In addition, ShA9 secretes AIPs that directly inhibit *S. aureus* Agr‐QS. In murine models, topical application of ShA9 alleviated dermatitis, and in a phase I clinical trial, a ShA9‐containing cream significantly reduced *S. aureus* colonization, restored microbial balance, and improved local eczema symptoms [85, 86]. Interestingly, even lantibiotic‐deficient ShA9 mutants retained therapeutic effects, supporting the importance of quorum quenching as a mechanism of action. In addition, a small clinical study reported that autologous transplantation of CoNS isolated from AD patients onto lesional skin competitively suppressed pathogenic *S. aureus* and improved dermatitis [86]. These findings suggest that commensals or compounds capable of competing with, or inhibiting, Agr‐QS‐dependent pathogenic strains represent a promising therapeutic option. However, such approaches remain at an experimental stage and require further refinement before translation into widespread clinical practice. Beyond commensal‐based strategies, other novel avenues are being explored. One approach focuses on precision antimicrobial therapy using bacteriophage endolysins, which are engineered to selectively target *S. aureus* while sparing other commensals. Preclinical and early translational studies support their potential as precision antimicrobial tools [87].

**FIGURE 4:**
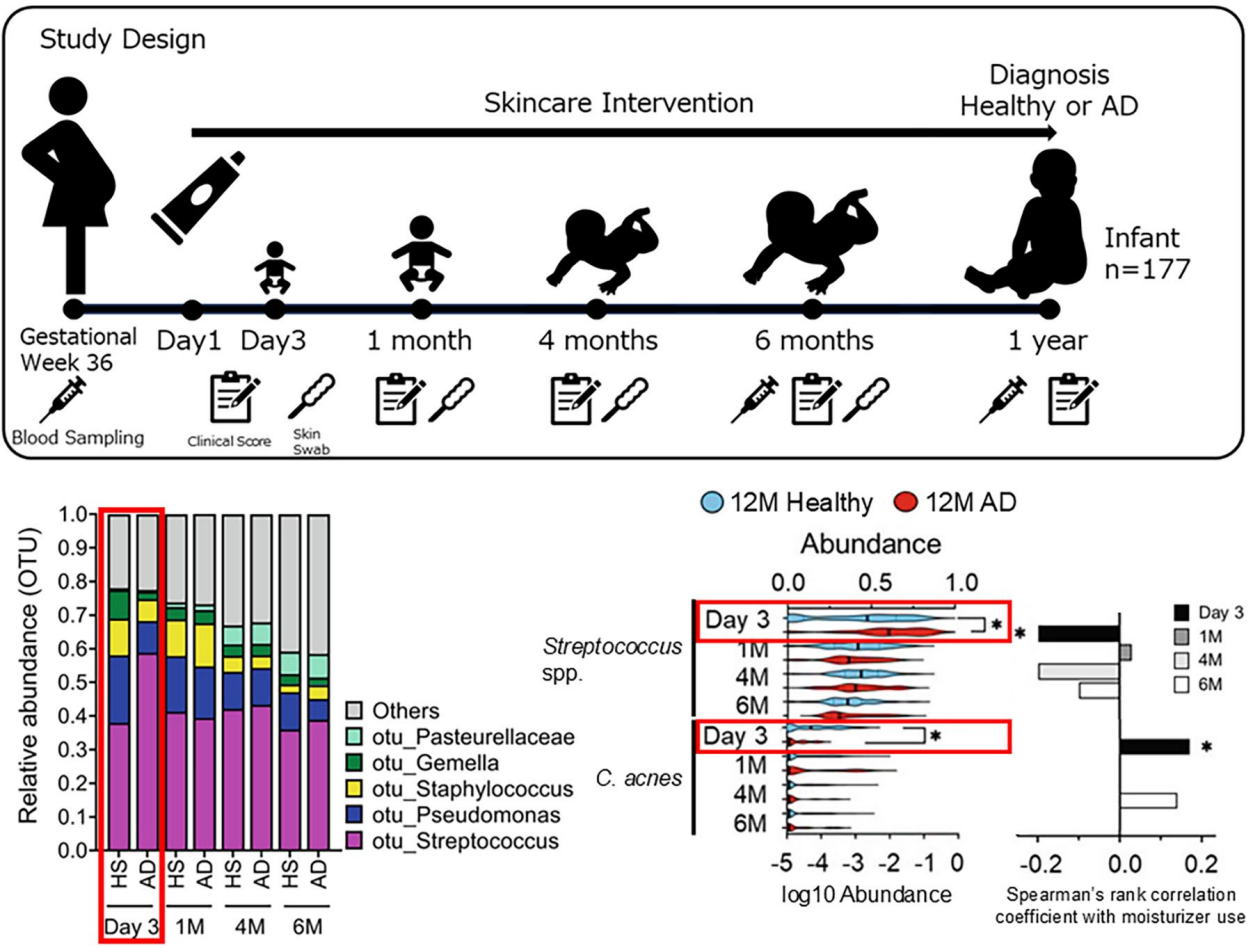
Neonatal skin dysbiosis associated with AD. In neonates who later develop AD by 1 year, skin *C. acnes* levels are decreased, while *Streptococcus* levels are increased. This early dysbiosis can be improved by the topical application of moisturizer. Early‐life skincare may thus act in a prebiotic‐like manner, supporting microbial balance and reducing AD risk. Figures and graphs are adapted from reference [90].

Primary prevention represents another critical frontier. Distinct strategies, such as maternal‐child microbial seeding interventions, are being explored in clinical trials to restore microbial communities in cesarean‐delivered infants, with the goal of reducing the risk of microbiome‐related immune disorders, including AD [88]. In parallel, preventive strategies focusing on early‐life skincare have also been evaluated. A randomized clinical study involving 321 participants demonstrated that daily emollient application initiated immediately after birth and continued until 2 months of age significantly reduced the incidence of AD during the first year of life in high‐risk infants [89]. Longitudinal skin microbiome analyses have provided mechanistic insights into these findings. In a Japanese neonatal cohort study, skin microbiome samples were collected longitudinally from birth and comparative analyses with clinical outcomes revealed distinct differences in microbial composition between healthy infants and those who developed AD by one year of age. Specifically, increased abundance of *Streptococcus* and *Prevotella* species and decreased abundance of *C. acnes* at day 3 of life were associated with subsequent AD onset. Moreover, the presence of *Streptococcus mitis* on neonatal skin was negatively correlated with moisturizer use, whereas *C*. *acnes* abundance was positively correlated with moisturizer application [90]. These findings suggest that early dysbiosis associated with AD onset may be ameliorated by appropriate use of moisturizers, which could act in a prebiotic‐like manner to reduce the risk of AD development (Figure 4).

Overall, these strategies represent a clear shift away from nonspecific eradication toward more precise modulation of the skin microbiome. Approaches such as commensal‐based bacteriotherapy, quorum‐quenching agents, phage‐derived endolysins, and early‐life skincare interventions all share a common goal: to preserve and restore a resilient microbial ecosystem rather than deplete it indiscriminately. Notably, evidence for emollient‐based primary prevention remains mixed across randomized trials, with several studies reporting neutral outcomes despite promising results in selected cohorts. Although most of these approaches are still in the preclinical or early clinical stages, they collectively demonstrate the growing feasibility of harnessing microbial balance as both a therapeutic and preventive strategy for AD.

## 10 Conclusion and Future Perspectives

The role of *S. aureus* in the pathogenesis of AD has prompted a significant shift in therapeutic development, with a growing focus on targeting the skin microbiome itself. Approaches that aim to restore the balance of the skin microbial ecosystem through strategies like leveraging commensal competition and implementing early skincare interventions have emerged as promising new avenues, representing a shift from simple pathogen eradication. For these strategies to be widely adopted in clinical practice, key questions must be addressed. Future research is required to elucidate the underlying mechanisms, clarifying how and why these therapies improve the skin environment. Furthermore, future studies are required to establish the long‐term safety and efficacy of these approaches, and to elucidate which patient populations will gain the most therapeutic benefit. Ultimately, a deeper understanding of the complex interplay between the skin microbiome and the host immune system is the key to unlocking the full potential of these novel therapeutic approaches for AD.

## Funding

This work was supported by Japan Society for the Promotion of Science (KAKENHI 23K27621). JST FOREST (JPMJFR200Y). AMED‐CREST (23gm1610004h0003). Japan Agency for Medical Research and Development (23ek0410105s0101). LEO Foundation award.

## Conflicts of Interest

The authors declare no conflicts of interest.

## Data Availability

Data sharing not applicable to this article as no datasets were generated or analyzed during the current study.
